# Leptin and Notch Signaling Cooperate in Sustaining Glioblastoma Multiforme Progression

**DOI:** 10.3390/biom10060886

**Published:** 2020-06-09

**Authors:** Salvatore Panza, Umberto Russo, Francesca Giordano, Antonella Leggio, Ines Barone, Daniela Bonofiglio, Luca Gelsomino, Rocco Malivindi, Francesca Luisa Conforti, Giuseppina Daniela Naimo, Cinzia Giordano, Stefania Catalano, Sebastiano Andò

**Affiliations:** 1 Department of Pharmacy, Health and Nutritional Sciences, University of Calabria, Via P. Bucci, 87036 Arcavacata di Rende (CS), Italy; sasapanza@libero.it (S.P.); umbrus@live.it (U.R.); francesca.giordano@unical.it (F.G.); antonella.leggio@unical.it (A.L.); ines.barone@unical.it (I.B.); daniela.bonofiglio@unical.it (D.B.); luca.gelsomino@unical.it (L.G.); rocco.malivindi@unical.it (R.M.); francescaluisa.conforti@unical.it (F.L.C.); giuseppinadaniela.naimo@unical.it (G.D.N.); cinzia.giordano@unical.it (C.G.); 2 Centro Sanitario, University of Calabria, Via P. Bucci, 87036 Arcavacata di Rende (CS), Italy

**Keywords:** glioblastoma multiforme, leptin, Notch

## Abstract

Glioblastoma multiforme (GBM) is the most malignant form of glioma, which represents one of the commonly occurring tumors of the central nervous system. Despite the continuous development of new clinical therapies against this malignancy, it still remains a deadly disease with very poor prognosis. Here, we demonstrated the existence of a biologically active interaction between leptin and Notch signaling pathways that sustains GBM development and progression. We found that the expression of leptin and its receptors was significantly higher in human glioblastoma cells, U-87 MG and T98G, than in a normal human glial cell line, SVG p12, and that activation of leptin signaling induced growth and motility in GBM cells. Interestingly, flow cytometry and real-time RT-PCR assays revealed that GBM cells, grown as neurospheres, displayed stem cell-like properties (CD133+) along with an enhanced expression of leptin receptors. Leptin treatment significantly increased the neurosphere forming efficiency, self-renewal capacity, and mRNA expression levels of the stemness markers CD133, Nestin, SOX2, and GFAP. Mechanistically, we evidenced a leptin-mediated upregulation of Notch 1 receptor and the activation of its downstream effectors and target molecules. Leptin-induced effects on U-87 MG and T98G cells were abrogated by the selective leptin antagonist, the peptide LDFI (Leu-Asp-Phe-Ile), as well as by the specific Notch signaling inhibitor, GSI (Gamma Secretase Inhibitor) and in the presence of a dominant-negative of mastermind-like-1. Overall, these findings demonstrate, for the first time, a functional interaction between leptin and Notch signaling in GBM, highlighting leptin/Notch crosstalk as a potential novel therapeutic target for GBM treatment.

## 1. Introduction

Glioblastoma multiforme (GBM) is the most common and aggressive primary intracranial malignancy, causing 3–4% of all cancer-related death [1,2,3]. Currently, the standard treatments for GBM include surgical resection in combination with radiotherapy and chemotherapy with Temozolomide [4]. However, despite enormous efforts in multimodal treatment approaches, the prognosis of glioblastoma remains very poor, with a median overall survival of less than 15 months after diagnosis [5]. GBM is characterized by extensive phenotypic, morphological, and cellular heterogeneity that is thought to be maintained by a population of transformed stem-like cells referred to as glioma stem cells (GSCs). It has been reported that GSCs contribute to tumor initiation, invasion, recurrence, and resistance to therapy due to their self-renewal ability and multi-lineage differentiation potential [6,7]. Thus, the identification of novel molecular mechanisms that sustain the stem-like properties of glioblastoma cells will be important for therapeutic purpose.

Growing evidence indicates that the adipokine leptin, a pleiotropic molecule encoded by the obese gene originally known for its role as a regulator of food intake and energy balance in the mammalian central nervous system, plays a crucial role in maintaining cancer in a stem-like state. Indeed, the expression of the leptin receptor (ObR) is a feature of cancer stem cells and its activation is able to regulate several signaling pathways and oncogenes, which are critically implicated in cancer stem cell activity [8,9]. It has been found that human GBM cell lines as well as human primary GBM tissues expressed leptin along with its receptor and their levels correlate with the degree of malignancy [10,11,12,13]. Furthermore, leptin was reported to enhance the migration and invasion of C6 rat glioblastoma cells mainly through the p38 mitogen-activated protein kinase (MAPK) and Nuclear Factor-kB pathway [14] to promote proliferation on human glioblastoma cell lines [15] and to exert pro-angiogenic effects [16].

Leptin is able to modulate the hallmarks of cancer development and progression, also through a functional interplay with other important molecular effectors, including estrogens, growth factors, and inflammatory cytokines [17,18,19,20,21,22]. In this context, the crosstalk between leptin and Notch pathways has been described in breast [23,24], pancreatic [25], and endometrial [26] cancers, where it affects cell proliferation, migration, invasion, angiogenesis, and chemoresistance. Notch signaling, an evolutionary conserved pathway, has been shown to play a key role in glioblastoma tumorigenesis [27]. Particularly, several studies have demonstrated that GSCs exhibit elevated Notch activity that contributes to suppress differentiation and to maintain stem cell-like properties, contributing to their conventional-treatment resistance [28,29,30,31,32]. Although the involvement of leptin and its receptors in GBM has been reported, the relationship between leptin and Notch signaling and their role in sustaining cancer stem cell activity in GBM remain to be completely unraveled.

In the present study, we show, for the first time, that leptin promotes proliferation, migration, and stemness of GBM cells through Notch signaling activation, opening the possibility to explore novel therapeutic strategies for the treatment of GBM.

## 2. Materials and Methods

### 2.1. Reagents, Antibodies, and Plasmids

Gamma Secretase Inhibitor (GSI) and AG490 were provided by Sigma Aldrich (Milan, Italy); human anti-β-Actin, anti-ObR, and anti-Ob antibodies from Santa Cruz Biotechnology (Santa Cruz, CA, USA); human anti-Janus-activated kinase (JAK)2, anti-signal transducer and activator of transcription 3 (STAT3), anti-pJAK2Tyr^1007/1008^, and anti-pSTAT3Tyr^705^ from Cell Signaling Technology (Beverly, MA, USA); and human anti-Prominin-1 (CD133) from Proteintech (Rosemont, IL, USA). The luciferase reporter plasmid HES1-Luc (−467 to +46 of Hair and Enhancer of Split 1 promoter with the luciferase gene), C promoter-binding factor 1 (CBF1)-Luc (10xCBF1-luciferase reporter), and the plasmid encoding a dominant-negative of mastermind-like-1 (DN-MAML-1) were provided by Prof. Maggiolini M. (University of Calabria, Italy) and Dr. Clarke R.B. (Breast Cancer Now Research Unit, Institute of Cancer Sciences, University of Manchester, UK), respectively.

### 2.2. Cell Culture

Human fetal glial cells SVG p12 and human glioblastoma cell lines U-87 MG and T98G were purchased from ATCC (Manassas, VA, USA). The SVG p12, U-87 MG, and T98G cells were cultured in Minimum Essential Medium (Life Technologies, Monza MB, Italy), including 10% heat-inactivated fetal bovine serum (FBS), 200 mM L-glutamine, 1% penicillin-streptomycin, 1% Eagle’s nonessential amino acids, and 1% sodium pyruvate (Sigma Aldrich). Cells were cultured at 37 °C in a humidified atmosphere with 5% carbon dioxide. Cells were stored following the supplier’s recommendations, and authenticated every six months after frozen aliquot resuscitations and regularly tested for mycoplasma negativity (MycoAlert Mycoplasma Detection Assay, Lonza, Basilea, CH, Switzerland).

### 2.3. Real-Time RT-PCR Assays

Analysis of gene expression was assessed by real-time RT-PCR, using SYBR Green Universal PCR Master Mix (Bio-Rad, Segrate, Italy) as previously described [33]. mRNA expression levels of different genes were normalized on GAPDH mRNA content. The relative gene expression levels were calculated as described [34]. Primers are listed in Table S1.

### 2.4. Immunofluorescence

Immunofluorescence microscopy analysis was carried out as previously reported [35]. Briefly, cells were incubated with anti-Ob, anti-ObR, and anti-CD133 antibodies (4 °C, overnight) followed by fluorescein isothiocyanate-conjugated secondary antibody (30 min at RT). IgG primary antibody was used as a negative control. 4’,6-Diamidino-2-phenylindole (DAPI; Sigma Aldrich) staining was used for nuclei detection. Fluorescence was photographed with an OLYMPUS BX51 microscope (×100 objective) (Tokyo, Japan).

### 2.5. Immunoblot Analysis

Protein extracts were subjected to SDS-PAGE as reported [36]. Immunoblots show a single representative of three separate experiments. The images were acquired using Odissey FC (Licor, Lincoln, NE, USA). The Scion Image laser densitometry scanning program was used to quantify the band of interest. Standard deviations along with associated *p* values for the biological replicates were determined by using the GraphPad-Prism7 software program (GraphPad Inc., San Diego, CA, USA).

### 2.6. [^3^H]Thymidine Incorporation

U-87 MG and T98G cells were treated as described for 24 h. For the last 6 h, [^3^H]Thymidine (1μCi/mL) was added to the culture medium, After incubation, cells were processed as previously described [37].

### 2.7. Wound Healing Assays

Cell monolayers were scraped and subjected to the different experimental conditions as indicated. Cell migration was monitored for 12 h and the rate of wound healing was quantified as reported [38]. Pictures represent one of three independent experiments (×10 magnification) (OLIMPUS-BX51 microscope).

### 2.8. Transmigration Assays

Cells treated as indicated were placed in the top compartment of a Boyden chamber (8-μm membranes; Corning Costar, Corning, NY, USA). The bottom well contained regular full media. After 12 h, migrated cells, fixed and stained with DAPI, were quantified by viewing 5 separate fields per membrane at ×10 magnification. Data are expressed as the mean number of migrated cells of three independent experiments, assayed in triplicate.

### 2.9. Neurosphere Culture

U-87 MG and T98G cells were enzymatically and manually disaggregated to obtain a single cell suspension, and were plated in ultra-low attachment plates (Corning Life Sciences) at a density of 200 cells/cm^2^ in a serum-free DMEM-F12 supplemented with B27, 1 mg/mL penicillin-streptomycin (Life Technologies), 20 ng/mL human epidermal growth factor (EGF, Sigma), 10 ng/mL basic fibroblast growth factor (FGF, PeproTech, London, UK), and 0.0002% heparin (Sigma). Leptin, GSI, LDFI, and AG490 were added at the beginning of the experiments. After 7 days, neurospheres ≥50 µm (primary neurospheres) were counted using a microscope (×10 magnification), collected, enzymatically dissociated, and plated at the same seeding density as in the primary generation. The neurosphere forming efficiency (NFE) was obtained by dividing the number of neurospheres formed (≥50 µm) by the number of seeded cells and is expressed as the mean percentage of NFE. Self-renewal (SR) was calculated by dividing the total number of secondary neurospheres formed/total number of primary neurospheres formed and reported as fold change vs. vehicle-treated cells.

### 2.10. Flow Cytometry

Cells were washed in PBS with 2.5% BSA and labeled with anti-human CD133 and incubated for 2 h at RT followed by incubation with fluorescein isothiocyanate (FITC)-conjugated secondary antibody (1 h at RT), according to the supplier’s protocol. Same-isotype irrelevant antibody was used as a negative control. Cells were analyzed using a FACScan flow cytometer and acquisition was analyzed with WinDI software (Becton Dickinson, Mountain View, CA, USA).

### 2.11. Soft Agar Assay

The soft agar anchorage-independent growth assay was assessed as described [39].

### 2.12. Limiting Dilution Assay (LDA)

The limiting dilution assay (LDA) was used to evaluate in vitro the frequency of self-renewing cells in our populations, following the protocol described by Seyfrid et al. [39]. Briefly, U-87 MG and T98G cells were grown as neurospheres, as previously described, and after 7 days of growth, neurospheres were dissociate into a single cell suspension and seeded in neurosphere culture media at decreasing densities (100, 50, 10, 1 cell/well) into 96-well ultra-low attachment plates. The frequency of GBSCs in the sample was determined by linear regression analysis using ELDA online software (http://bioinf.wehi.edu.au/software/elda/) [40]. Data were displayed as a scatter plot graph showing in the Y-axis the percentage of wells without detectable spheres, and on the X-axis the number of the seeded cells/well.

### 2.13. Transient Transfection Assays

Cells were transfected with 1 µg of luciferase reported plasmid HES1-Luc (−467 to +46 of the promoter with the luciferase gene) or CFB1-Luc (10xCBF1-luciferase reporter) and 20 ng of TK Renilla Luciferase plasmid was used as an internal control using lipofectamine reagent (Life Technologies). After 6 h, treatments were added as indicated and cells were incubated for 12 h. Luciferase activity was measured as reported [41]. The normalized relative light unit values obtained from cells treated with vehicle were set as one-fold induction upon which the activity induced by treatments was calculated. In a set of experiments, cells were transiently transfected with a plasmid encoding an empty vector (e.v.) or a plasmid encoding for the dominant-negative of MAML-1 (DN-MAML-1, 5 µg), and after 24 h, transfected cells were plated as neurospheres to evaluate NFE.

### 2.14. Statistical Analysis

Each datum point represents the mean ± standard deviation (SD) of three different experiments. Data were analyzed for statistical significance (*p* < 0.05) using a two-tailed student’s Test, performed by GraphPad-Prism7 software program (GraphPad Inc., San Diego, CA, USA).

## 3. Results

### 3.1. Leptin Induces Growth and Migration of GBM Cell Lines

**Figure 1:**
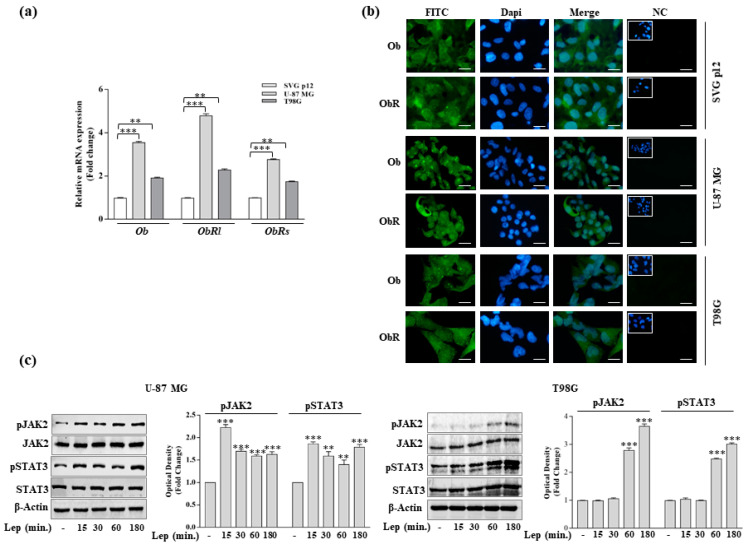
Leptin and leptin receptor expression and signaling activation in GBM cells. (**a**) Real-time RT-PCR for *Ob* and *ObR* long and short isoforms (*ObRl* and *ObRs,* respectively) in U-87 MG and T98G cells; mRNA expression is shown relative to SVG p12 normal human glial cells. (**b**) Immunofluorescence of Ob and ObR in U-87 MG, T98G, and SVG p12 cells. DAPI staining for nuclear detection. Scale bars = 5 µm. Original magnification, ×100. FITC, fluorescein isothiocyanate; NC, negative control. (**c**) Levels of phosphorylated (p) JAK2 and (p) STAT3 and relative total proteins evaluated by immunoblotting in total extracts from cells treated with vehicle (-) or Leptin (Lep, 500 ng/mL) as indicated. β-Actin was used as a loading control. The histograms represent the means ± SD of three separate experiments in which band intensities were evaluated in terms of optical density arbitrary units and expressed as a fold change in phospho-proteins/total proteins/β-Actin vs. vehicle-treated samples, which were assumed to be 1. ** *p* < 0.01, and *** *p* < 0.001.

We first aimed to evaluate the expression of leptin (Ob) and its receptor (ObR) in a normal human glial cell line (SVG p12) and in two GBM cell lines (U-87 MG and T98G) by real-time RT-PCR analysis. Compared to normal cell line, expression levels of *Ob* as well as the long (*ObRl*) and the short (*ObRs*) isoforms of the leptin receptor were significantly upregulated in both U-87 MG and T98G cells (Figure 1a). These data were also confirmed at the protein level by using immunofluorescent staining (Figure 1b). The essential initial event triggered by leptin binding to its receptor is JAK/STAT signaling activation. Time course–response studies evidenced in both GBM cell lines a significant increase in the phosphorylation status of JAK2 and STAT3 after leptin treatment (500 ng/mL) (Figure 1c). Thus, in agreement with previous results [10,11,12,13], we found that GBM cell lines express Ob and a functional ObR.

**Figure 2:**
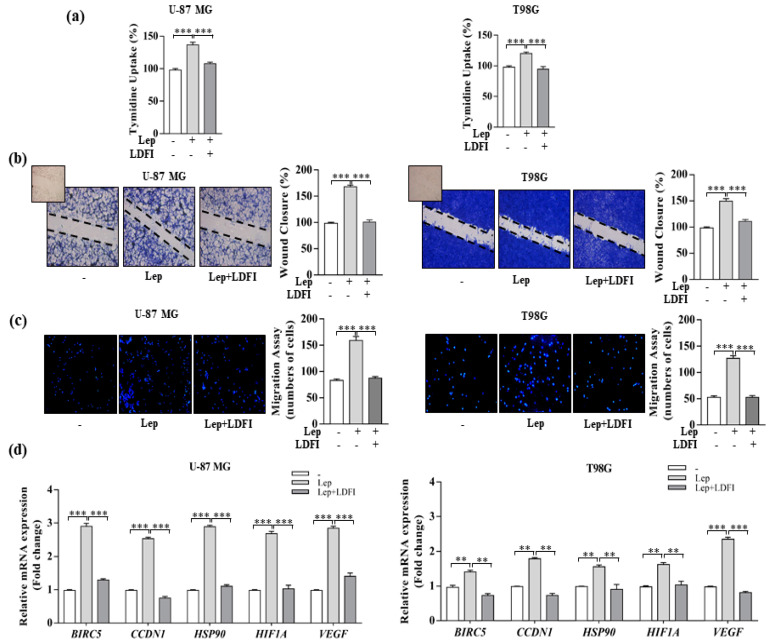
Effects of a selective leptin receptor antagonist peptide LDFI on leptin-induced U-87 MG and T98G cell proliferation and migration. (**a**) Cell proliferation was determined by the ^[3H]^thymidine incorporation assay in cells treated with vehicle (-) and leptin (Lep, 500 ng/mL) alone or in combination with the leptin antagonist (LDFI, 1 µmol/L) for 24 h. (**b**) Wound healing assays in U-87 MG and T98G cells treated for 12 h with vehicle (-) and leptin (Lep, 500 ng/mL) alone or in combination with the leptin antagonist (LDFI, 1 µmol/L). Images are representative of three independent experiments. The histograms represent the relative percentage of wound closure calculated by ImageJ software version 1.51q. Small squares, time 0. Original magnification, ×10. (**c**) Boyden chamber transmigration assays in U-87 MG and T98G cells treated with vehicle (-) and leptin (Lep, 500 ng/mL) alone or in combination with the leptin antagonist (LDFI, 1 µmol/L) for 12 h. A representative image of transmigrated cells is shown. Data are expressed as means ± SD of three different experiments, each performed in triplicate. Original magnification, ×10. (**d**) Real-time RT-PCR for mRNA levels of a subset of leptin-target genes in U-87 MG and T98G cells treated for 24 h with vehicle (-) and leptin (Lep, 500 ng/mL) alone or in combination with the leptin antagonist (LDFI, 1 µmol/L). Data are expressed as means ± SD of three different experiments, each performed in triplicate. ** *p* < 0.01; *** *p* < 0.001.

Then, we examined the effects of leptin on the proliferation and motility of GBM cells. Results from the thymidine incorporation assay showed that leptin significantly enhanced the proliferation of both U-87 MG and T98G cells after 24 h of treatment compared with the vehicle-treated cells (Figure 2a). Moreover, leptin exposure increased cell migration in both wound healing (Figure 2b) and transmigration (Figure 2c) assays, suggesting that this cytokine could facilitate the invasive behavior of GBM cells. The direct involvement of leptin/leptin receptor-mediated signaling in the observed effects was confirmed by using a selective leptin receptor antagonist, the peptide LDFI, a small peptide of the wild-type sequence of the leptin binding site I (Leu-Asp-Phe-Ile), synthesized by solid phase methodologies, which we previously demonstrated to specifically inhibit both the in vitro and in vivo leptin signaling pathway [8,37,42,43]. 

In the presence of LDFI peptide, the leptin-mediated effects on cell growth and motility were completely reversed (Figure 2a–c). In addition, the ObR antagonist LDFI strongly reduced leptin induction on the expression levels of the well-known leptin target genes involved in cell proliferation and progression [8,37,42,43], including survivin (*BIRC5*), cyclin D1 (*CCDN1*), *HSP90A*, *HIF1A*, and *VEGF* (Figure 2d).

All these data highlight a crucial role for an activated leptin/leptin receptor axis in promoting growth and migration of GBM cells.

### 3.2. Leptin Promotes the Stemness of Glioblastoma Cells

**Figure 3:**
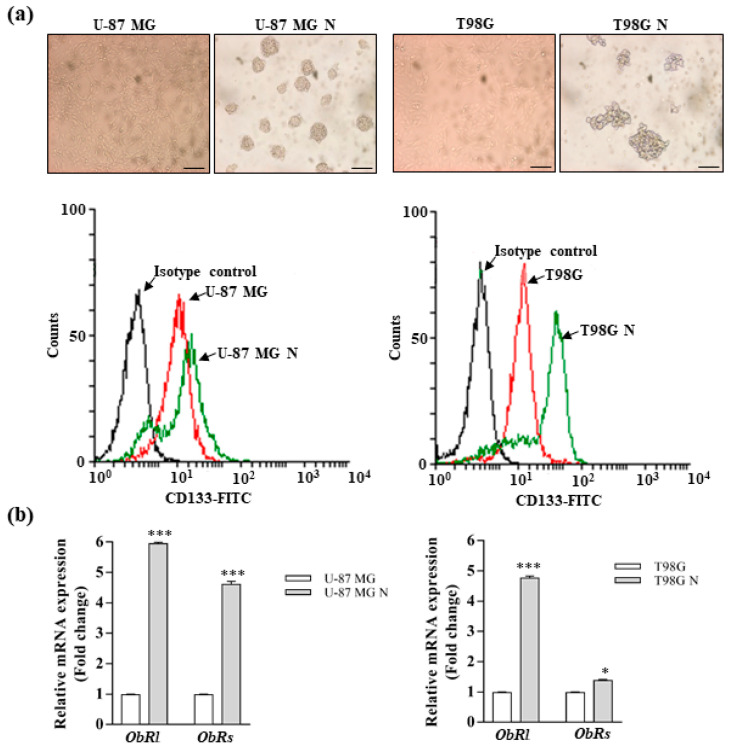
Characterization of glioblastoma stem-like cells. (**a**) Representative phase contrast images of neurospheres derived from U-87 MG and T98G cells (U-87 MG N and T98G N, respectively). Upper panel. Scale bars = 50 µm. Original magnification, ×10. Lower panel. Flow cytometry analysis of the CD133-positive (CD133-FITC) subpopulation of cells growth as neurospheres. (**b**) Leptin receptor long (*ObRl*) and short (*ObRs*) isoform mRNA levels, evaluated by real-time RT-PCR, in U-87 MG, U-87 MG N, T98G, and T98G N cells. Data are expressed as means ± SD of three different experiments, each performed in triplicate. * *p* < 0.05 and *** *p* < 0.001.

Since it has been reported that the leptin receptor plays a crucial role in maintaining cancers in a stem cell-like state [8,44,45,46], we investigated the effects of this cytokine on the stemness of GBM cells. Thus, we established the neurosphere cultures to enrich glioma stem-like cells (GSCs) from U-87 MG and T98G cell lines (Figure 3a, upper panel) and characterized the obtained cell population by flow cytometric analysis to evaluate the enrichment of neurospheres in GSC subpopulations expressing the CD133 marker (CD133+). As expected, we detected an enrichment of the CD133+ subpopulation in cells grown as neurospheres from both cells lines with respect to monolayer cells (Figure 3a, lower panel). Interestingly, significantly higher mRNA levels of both *ObRs* and *ObRl* isoforms were observed in the cell growth as neurospheres compared with the corresponding monolayer cell cultures (Figure 3b).

**Figure 4:**
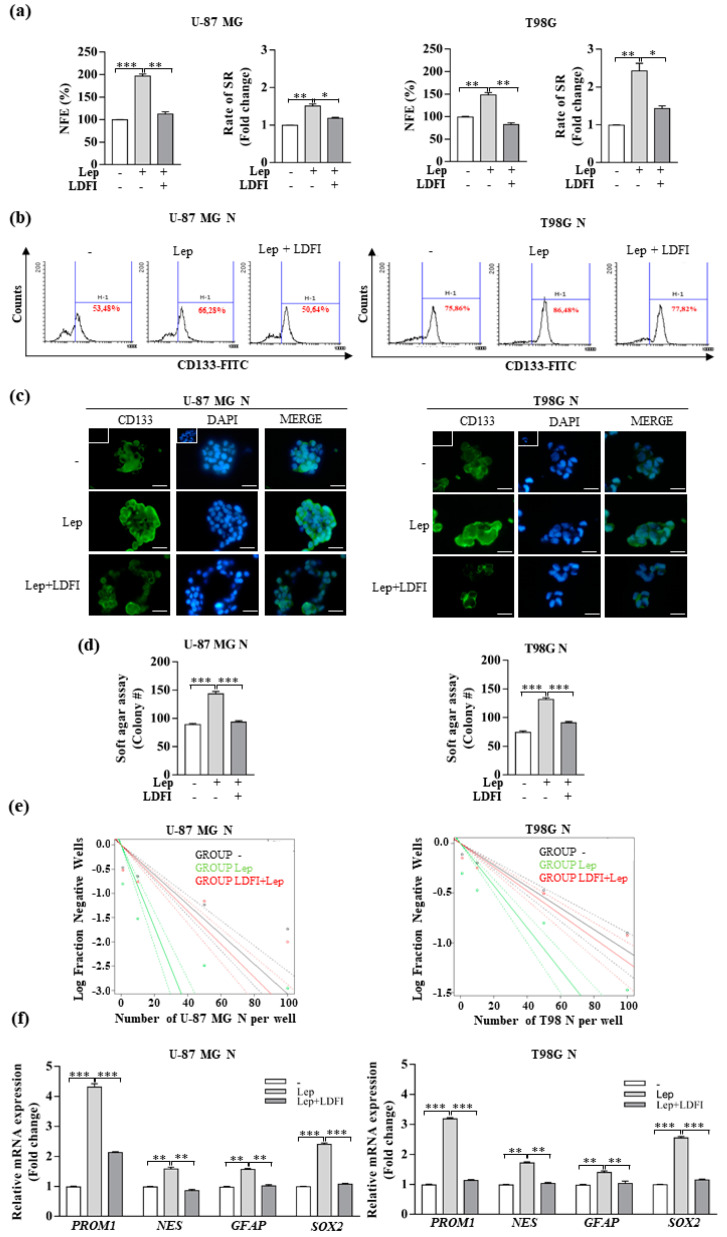
Effects of leptin on glioblastoma stem-like cell activity. (**a**) NFE and SR in U-87 MG and T98G cells cultured as neurospheres (U-87 MG N and T98G N, respectively) in the presence of vehicle (-) or leptin (Lep, 500 ng/mL) alone or in combination with the leptin antagonist (LDFI, 1 µmol/L). (**b**) Flow cytometry analysis of CD133-FITC expression in U-87 MG N and T98G N cultured in the presence of vehicle (-) or leptin (Lep, 500 ng/mL) alone or in combination with LDFI, 1 µmol/L. (**c**) Immunofluorescence of CD133 expression in U-87 MG N and T98G N cultured as reported. DAPI staining for nuclear detection. Scale bars = 5 µm. Original magnification, ×100. FITC, fluorescein isothiocyanate; Small squares, negative control. (**d**) U-87 MG N and T98G N cells were plated in soft agar as described in the materials and methods. After 14 days of growth, colonies >50 µm were counted. The histogram represents the mean ± SD of two different experiments. (**e**) GBM self-renewal capacity was assessed by the limiting dilution assay (LDA) in U-87 MG N and the T98G N. The dotted line plots show a representative linear regression plot of LDA for U-87 MG and T98G cells treated with the vehicle (-, black) or leptin (Lep, 500ng/mL, red) in the presence or not of LDFI (1µmol/L). After 14 days of growth, neurosphere formation was evaluated. The dashed lines give the 95% confidence interval, calculated with the ELDA software. (**f**) Real-time RT-PCR for a subset of genes associated with the stemness phenotype in U-87 MG N and T98G N cultured in the presence of the vehicle (-) or leptin (Lep, 500 ng/mL) alone or in combination with LDFI, 1 µmol/L. Data are expressed as means ± SD of three different experiments, each performed in triplicate. * *p* < 0.05, ** *p* < 0.01, and *** *p* < 0.001.

Accordingly, leptin treatment in GBM cells significantly enhanced the sphere-forming efficiency as well as self-renewal (Figure 4a) and increased the CD133+ subpopulation (Figure 4b,c). As expected, the addition of the ObR antagonist LDFI significantly decreased these effects (Figure 4a–c). In addition, to further confirm the enhanced clonogenic potential and self-renewal properties of glioblastoma cells treated with leptin, we performed the soft agar assay and the limiting dilution assay (LDA), following the methods previously described [39]. As shown in Figure 4d, leptin treatment significantly increased colony formation in the anchorage-independent soft agar assay. Moreover, the self-renewing frequencies of GBM stem cells, calculated using ELDA software, showed significant differences between the vehicle- and leptin-treated groups (Figure 4e and Figure S2a–c). The change of the slope of the trend line in the leptin-exposed cells suggested a differential neurosphere formation ability as indicated by the increase in the stemness frequency of leptin-treated U-87 MG and T98G cells (1/11.8 and 1/47.1, respectively) compared to untreated cells (1/32.5 and 1/92.4, respectively) (Figure S2b,c). As expected, the presence of the LDFI peptide abrogated leptin-induced effects in both soft agar and LDA assays (Figure 4d,e and Figure S2a–c). Real-time RT-PCR also revealed that leptin increased the mRNA levels of stemness markers, including CD133 *(PROM1)*, Nestin *(NES)*, *GFAP*, and *SOX2*, and this upregulation was completely reversed in the presence of LDFI (Figure 4d). Finally, the involvement of leptin signaling in inducing stemness was also demonstrated by using a specific JAK2/STAT3 inhibitor (AG490). Particularly, we assessed the NFE and soft agar assay in GMB cells treated with leptin in the presence or not of AG490. As reported in Figure S1a,b, the presence of AG490 reversed leptin’s effects on GMB stem cell activity.

Collectively, these results suggest that ObR activity may sustain the cancer stem-like properties in GBM cells.

### 3.3. Leptin Induces Notch Expression in GBM Cells

**Figure 5:**
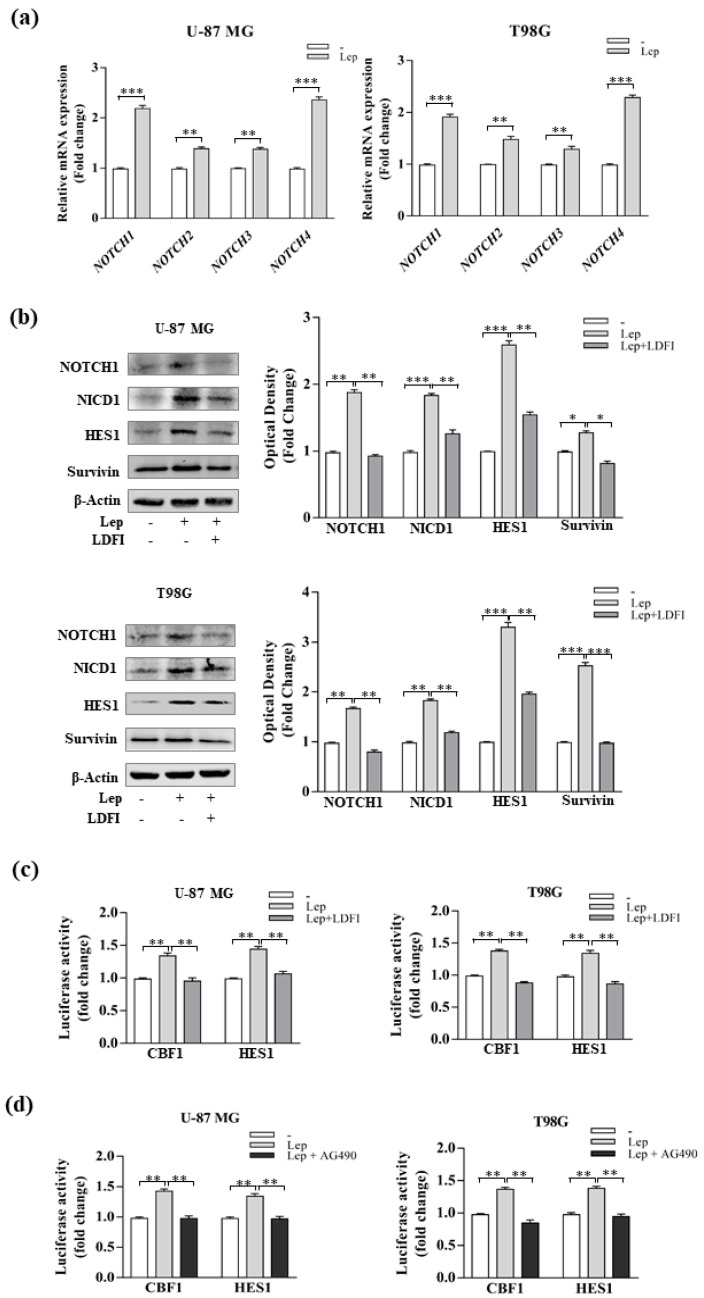
Leptin induces the expression of NOTCH and its molecular targets in glioblastoma cells. (**a**) Real-time RT-PCR for *NOTCH1, NOTCH2, NOTCH3,* and *NOTCH4* receptors in U-87 MG and T98G cells treated for 12 h with vehicle (-) or 500 ng/mL leptin (Lep). (**b**) Expression levels of NOTCH1, NICD1, HES1, and Survivin, in U-87 MG and T98G cells treated with vehicle (-) and leptin (Lep, 500 ng/mL) alone or in combination with LDFI, 1 µmol/L for 12 h. β-Actin was used as a loading control. The histograms represent the means ± SD of three separate experiments in which band intensities were evaluated in terms of optical density arbitrary units and expressed as a fold change vs. vehicle-treated samples, which were assumed to be 1. U-87 MG and T98G cells transiently transfected with CBF1-Luc or HES1-Luc reporter constructs. After transfection, cells were treated with vehicle (-), and leptin (Lep, 500 ng/mL) alone or in combination with LDFI, 1 µmol/L (**c**), or treated with vehicle (-), and leptin (Lep, 500 ng/mL) alone or in combination with AG490, 20 µmol/L for 12 h (**d**). The histograms represent the means ± SD of three separate experiments, each performed in triplicate. * *p* < 0.05, ** *p* < 0.01, and *** *p* < 0.001.

To explore the underlying mechanisms of leptin in regulating stem-like traits, we first evaluated the Notch signaling pathway in GBM cells treated with leptin. As the Notch receptor family contains four isoforms, including *NOTCH1*, *NOTCH2*, *NOTCH3*, and *NOTCH4*, the mRNA levels of all four isoforms were examined in GBM cells by real-time RT-PCR. As shown in Figure 5a, leptin significantly increased the expression of these genes in GBM cells.

The Notch1-mediated signaling pathway plays a crucial role in the maintenance of neural stem cells and is critical for glioma cell growth and progression [47,48,49]. Immunoblot analysis revealed an upregulation of Notch1 full-length and its intracellular domain (NICD1) after leptin treatment, suggesting that Notch1 activation may play a critical role in leptin-induced enrichment of CSCs (Figure 5b). Moreover, leptin-treated cells had increased expression of Notch target genes, including HES1 and survivin (Figure 5b). The LDFI peptide decreased leptin’s effects (Figure 5b), indicating the specificity of leptin-induced changes on the levels of Notch components.

Consistent with the above results, leptin-treated cells showed enhanced Notch transcriptional activity, evaluated by the CBF1-Luc reporter gene, as well as HES1 transcriptional activity compared with untreated cells (Figure 5c). Interestingly, Notch transcriptional activity was completely reversed in the presence of LDFI (Figure 5c), as well as by using the JAK2/STAT3 inhibitor AG490 (Figure 5d). These latter results strongly support the hypothesis that Notch pathway activation could be modulated by leptin/leptin receptor interaction in GBM cells.

### 3.4. Interference of Notch Activation Reduces Leptin Effects on Growth and Progression of GBM Cells

**Figure 6:**
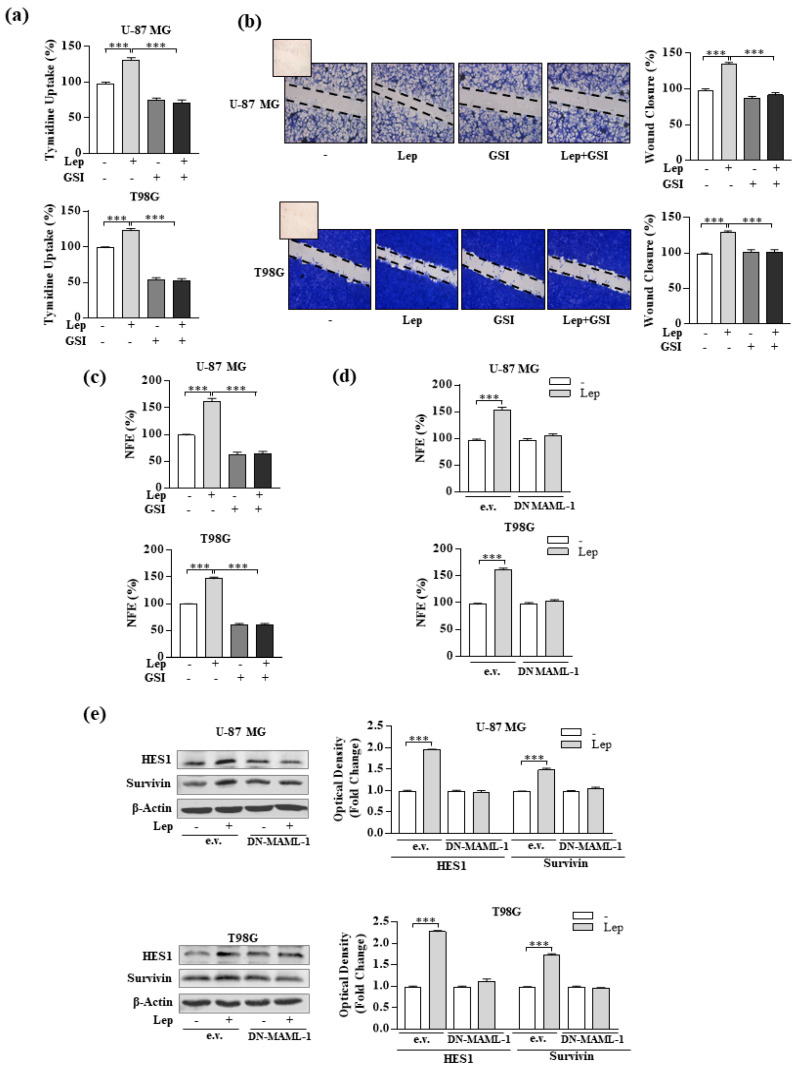
Effects of Notch inhibition on leptin-induced cells’ proliferation, migration, and stem cell activity in GBM cells. (**a**) Cell proliferation was determined by the ^[3H]^thymidine incorporation assay after 24 h of treatment with vehicle (-), leptin (Lep, 500 ng/mL), and γ-secretase inhibitor (GSI, 1 µmol/L) alone or in combination. (**b**) Wound healing assays in U-87 MG and T98G cells treated for 12 h with vehicle (-), leptin (Lep, 500 ng/mL), and γ-secretase inhibitor (GSI, 1 µmol/L) alone or in combination. Images are representative of three independent experiments. The histograms represent the relative percentage of wound closure calculated by ImageJ software version 1.51q. Small squares, time 0. Original magnification, ×10. (**c**) NFE in U-87 MG and T98G cells cultured in the presence of vehicle (-), leptin (Lep, 500 ng/mL), and γ-secretase inhibitor (GSI, 1 µmol/L) alone or in combination. (**d**) NFE in U-87MG and T98G cells transiently transfected with an empty vector (e.v.) or the DN-MAML-1 plasmid, and treated with vehicle (-) or leptin (Lep, 500 ng/mL). (**e**) Expression levels of HES1 and survivin, in U-87 MG and T98G cells transiently transfected with an empty vector (e.v.) or DN-MAML-1 for 24 h and then treated with vehicle (-) or leptin (Lep, 500 ng/mL) for 12 h. β-Actin was used as the loading control. The histograms represent the means ± SD of three separate experiments in which band intensities were evaluated in terms of optical density arbitrary units and expressed as a fold change vs. vehicle-treated samples, which were assumed to be 1. The histograms represent the means ± SD of three separate experiments, each performed in triplicate. *** *p* < 0.001.

To investigate whether targeted inhibition of the Notch signaling pathway could reverse the reported induction of cell proliferation and migration mediated by leptin, we treated cells with a Notch inhibitor (RO4929097, GSI), a potent and selective small molecule inhibitor of gamma-secretase that is currently in phase 1 of clinical trials in patients with glioma [50]. As shown in Figure 6a–c, leptin effects on cell proliferation, migration, and neurosphere formation were completely abrogated by inhibition of Notch signaling with GSI in both U-87 MG and T98G cell lines.

Furthermore, we performed experiments in glioblastoma cells transiently transfected with a plasmid encoding a dominant-negative mutant of the mastermind-like-1 (DN-MAML-1), an essential Notch nuclear co-activator involved in transcriptional events that are specific to the Notch pathway [51,52]. Particularly, we evaluated leptin’s effects on NFE and protein expression of the Notch target genes HES1 and survivin in cells expressing the DN-MAML-1. As expected, in the presence of DN-MAML-1, leptin induction was abrogated, confirming the involvement of Notch signaling in leptin-mediated actions in glioblastoma cells (Figure 6d,e, respectively).

Collectively, these data suggest that the Notch signaling pathway could be engaged with the activated leptin signaling in sustaining glioblastoma growth and progression.

## 4. Discussion

Glioblastoma multiforme (GBM) is the most common malignant primary brain tumor, featuring extreme aggressiveness, treatment resistance, and tumor relapse. Despite advances in treatment modalities, to date, GBM remains largely incurable due to its heterogeneity, plasticity, and complex pathogenesis [53,54]. Therefore, advances in the understanding GBM biology are urgently needed to discover new potential therapeutic targets. Here, we identified a novel leptin-mediated mechanism that promotes glioblastoma progression. We found that leptin could contribute to the development of GBM through mechanisms involving the leptin–Notch axis that increase the proliferation, migration, and expansion of GSC populations.

A growing body of evidence has raised the important role of leptin, an adipokine mainly produced by adipocytes but also by epithelial tumor cells itself and by other stroma cells, as an active player involved in cancer development and progression [17]. Indeed, data from clinical and experimental studies have demonstrated that leptin, through its binding to the transmembrane leptin receptor, induces the activation of many intracellular signal pathways involved in the control of cell proliferation, differentiation, survival, migration, and invasion [18,37,55]. Leptin and its receptor are overexpressed in cancer tissues compared with healthy epithelium and benign lesions and were found to be associated with a higher incidence, increased progression, and poor prognosis of several human cancers [56,57,58].

In line with previous findings [11,12], we demonstrated that the expression of leptin and its receptor were significantly higher in GBM cells than in human normal glial cells. Moreover, leptin treatment induced an activation of its intracellular signaling and significantly promoted the proliferation and migration of GBM cells. Targeting leptin signaling, by a selective leptin receptor antagonist peptide LDFI, completely reversed the leptin-mediated effects on cell growth and motility as well as reduced the expression of several leptin-induced target genes, confirming the relevant role of this adipokine in the biology of GBM.

The aggressive features of GBM is considered to result, at least in part, from the expansion of cancer stem cell populations with self-renewal and differentiation abilities, which are responsible for tumor growth, drug resistance, and tumor relapse [6]. During the last 10 years, the role of leptin in promoting the expansion of cancer stem cells has been reported in breast and other cancers [8,58,59,60]. It has also been demonstrated that leptin enhances the invasive potential of glioma stem-like cells [10], and high expression of ObR leads to temozolomide resistance through increased stem/progenitor cell features [12]. Our results extended these previous findings by demonstrating a direct involvement of leptin in sustaining cancer stem-like properties in GBM cells. We found that tumor spheres from GBM cells exhibited increased levels of the leptin receptor and treatment with leptin induced an increase in the neurosphere forming efficiency and self-renewal capacity, in the clonogenic potential and stem cell frequency, along with an enrichment of the CD133+ cell population. Interestingly, the addition of the selective ObR antagonist LDFI and the JAK2/STAT3 inhibitor AG490 significantly decreased these effects.

Leptin functions are strengthened through cross talk with other different signaling molecules, including estrogen receptor α (ERα) [19], growth factors [20,21], inflammatory cytokines [22], and Notch [23,24,25,26,61].

Notch is an important signal transduction pathway that plays a critical role in various cellular and developmental processes, including stem cell maintenance. In mammals, four Notch receptors (Notch1-4) and five Notch ligands (JAG1 and 2, Delta-Like (DDL)1, DLL3, and DLL4) have been identified [62]. The interaction between Notch and its ligands triggers enzymatic cleavage that liberates the Notch intracellular domain (NICD), which translocates to the nucleus and binds CBF1/suppressor of hairless/Lag-1 or CSL to activate its target genes [63]. Notch signaling has been shown to regulate neural stem cells and GSCs during normal neurogenesis and pathological carcinogenesis, respectively [27]. A higher expression of various Notch components and its correlation with a higher glioma grade and a worse prognosis has been reported [64,65,66]. Particularly, previous investigations indicated that *NOTCH1* and its target gene *HES1* were markedly increased in GSCs, leading to tumor invasion and recurrence of GBM [67,68,69], and Notch1 overexpression was also associated with low overall survival [70].

Our data showed, for the first time, a leptin-mediated upregulation of *NOTCH1* expression and transcriptional activity in GBM cells. Targeting leptin signaling by using the peptide LDFI, or blocking JAK2/STAT3 signaling completely abrogated these effects, suggesting that the possibility that pharmacologically blocking leptin signaling might attenuate Notch activity in GBM. This issue should be carefully addressed by further in vivo studies.

To date, several classes of Notch inhibitors have been developed. The most employed in cancer are γ-secretase inhibitors (GSIs), which prevent the release of active NICD from the receptor by the γ-secretase complex. In accordance with the above reported data, we demonstrated that treatment with GSI decreased leptin-induced growth, migration, and stemness in GBM cells. Moreover, the expression of DN-MAML-1, an essential nuclear co-activator involved in Notch transcriptional events, disturbed leptin effects on NFE and Notch target gene expression, suggesting that leptin-mediated effects in GBM might be sustained by the Notch signaling pathway.

## 5. Conclusions

GBM is characterized by a higher degree of invasiveness, resulting in a lethal primary malignancy. Many findings suggest that the main cause of the lack of success in GBM treatments rely on a subpopulation of high tumorigenic glioma stem cells (GSCs) able to promote tumor growth and progression, and to overcome radiotherapy and chemotherapy effects. Indeed, although during the last decades several innovations have been introduced in GBM treatment, their effectiveness remains limited, and treatment of this malignant disease has remained a challenge. Here, we reported findings highlighting the important role of Notch signaling in sustaining leptin-induced GBM progression, through enhanced GSC activity. These data, in addition to shedding light on the functions of the adipokine leptin and providing new insights into the regulation of the Notch pathway in GBM, suggest that targeting leptin–Notch signaling interaction could be a potential novel therapeutic option to treat GBM.

## Supplementary Materials

The following are available online at https://www.mdpi.com/2218-273X/10/6/886/s1, Table S1: Oligonucleotide primers used in this study. Figure S1. Effects of the JAK2/STAT3 inhibitor AG490 on leptin-induced NFE and anchorage-independent growth. Figure S2. Analysis of self-renewal using the extreme limiting dilution assay (ELDA) tool in U-87 MG N and T98G N cells.
