# Supplementary material for: Leptin and Notch Signaling Cooperate in Sustaining Glioblastoma Multiforme Progression
Source: Biomolecules. 2020 Jun 9;10(6):886. doi: 10.3390/biom10060886 (PMC7356667; doi:10.3390/biom10060886)
Supplement: Supplementary file 1 [file biomolecules-10-00886-s001.zip › Figure S1.pdf]

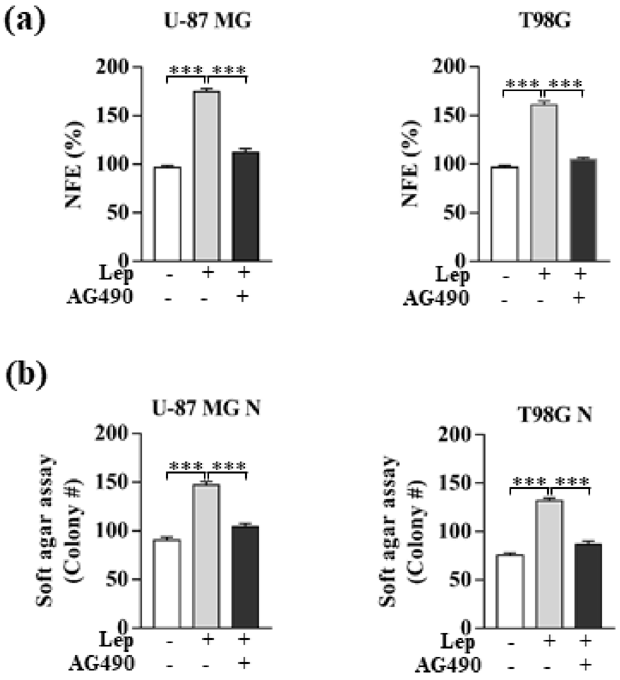

Figure S1. Effects of the JAK2/STAT3 inhibitor AG490 on leptin-induced NFE and anchorage-independent growth. (a) NFE in U-87 MG and T98G cells cultured as neurospheres (U-87 MG N and T98G N, respectively) in the presence of vehicle (-) or Leptin (Lep, 500 ng/mL) alone or in combination with the JAK/STAT3 inhibitor AG490 (20 μ M). (**b**) U-87 MG N and T98G N cells were plated in soft agar as described in Materials and Methods. After 14 days of growth, colonies >50 μm were counted. The histogram represent the mean ± SD of two different experiments. The histogram represents the means ± SD of two separate experiments, each performed in triplicate. ***P < 0.001.
