# Supplementary material for: Leptin and Notch Signaling Cooperate in Sustaining Glioblastoma Multiforme Progression
Source: Biomolecules. 2020 Jun 9;10(6):886. doi: 10.3390/biom10060886 (PMC7356667; doi:10.3390/biom10060886)
Supplement: Supplementary file 1 [file biomolecules-10-00886-s001.zip › Figure S2.pdf]

(a)

U-87 MG N

Counter	Dose	Tested	Response	Group
1	1	96	36	-
2	10	96	46	-
3	50	96	68	-
4	100	96	79	-
5	1	96	53	Lep
6	10	96	75	Lep
7	50	96	88	Lep
8	100	96	91	Lep
9	1	96	39	LDFI+Lep
10	10	96	51	LDFI+Lep
11	50	96	66	LDFI+Lep
12	100	96	83	LDFI+Lep

T98G N

Counter	Dose	Tested	Response	Group
1	1	96	10	-
2	10	96	17	-
3	50	96	36	-
4	100	96	57	-
5	1	96	25	Lep
6	10	96	36	Lep
7	50	96	53	Lep
8	100	96	74	Lep
9	1	96	13	LDFI+Lep
10	10	96	21	LDFI+Lep
11	50	96	38	LDFI+Lep
12	100	96	58	LDFI+Lep

(b)

Group	Lower	Estimate	Upper
-	38.5	32.5	27.55
LDFI+Lep	34.7	29.3	24.77
Lep	14.5	11.8	9.63

Group	Lower	Estimate	Upper
-	111.5	92.4	76.6
LDFI+Lep	100.6	83.8	69.8
Lep	55.7	47.1	39.9

(c)

Chisq	DF	P.value
96.5	2	1.14e-21

Chisq	DF	P.value
37	2	9.15e-09

Figure S2. Analysis of self-renewal using the extreme limiting dilution assay (ELDA) tool in U-87 MG N and T98G N cells. (**a**) Data entered in ELDA software to test differences in stem cell frequencies among groups. (**b**) Confidence intervals for 1/(stem cell frequency) in the vehicle-, Leptin (Lep 500 ng/ml)- and Lep+LDFI (1μmol/L)-treated groups. (**c**) Test for differences in stem cell frequencies among the three tested groups.
