# Supplementary material for: Leptin and Notch Signaling Cooperate in Sustaining Glioblastoma Multiforme Progression
Source: Biomolecules. 2020 Jun 9;10(6):886. doi: 10.3390/biom10060886 (PMC7356667; doi:10.3390/biom10060886)
Supplement: Supplementary file 1 [file biomolecules-10-00886-s001.zip › Table S1.pdf]

Table S1: Oligonucleotide primers used in this study

Gene Name	*Gene Symbol*	*Primers Sequences*
Leptin	*OB*	FOR 5′- GTGCGGATTCTTGTGGCTTT-3′<br>REV 5′- GGAATGAAGTCCAAACCGGTG-3′
Long form Leptin Receptor	*OBRl*	FOR 5′- TCACTCCGAAAGCAACAGTG-3′<br>REV 5′- CTTTTGCCTGCTGGACTCTC-3′
Short form Leptin Receptor	*OBRs*	FOR 5′- ATTGTGCCAGTAATTATTTCCTCTTCC-3′<br>REV 5′- CCACCATATGTTAACTCTCAGAAGTTCAA-3′
Cyclin D1	*CCND1*	FOR 5′- GATGCCAACCTCCTCAACGAC-3′<br>REV 5′- CTCCTCGCACTTCTGTTCCTC-3′
Heat Shock Protein 90	*HSP90A*	FOR 5′- GGGTAAAAGTAGAGAGAAGGATCAAGGA-3′<br>REV 5′- TGCATTTAATCCATCCAACTGAA-3′
Vascular endothelial growth factor	*VEGF*	FOR 5′- CCTGGTGGACATCTTCCAGGA-3′<br>REV 5′- CTCACCGCCTCGGCTTGTCAC-3′
Hypoxia-inducible factor 1-alpha	*HIF1A*	FOR 5′- TGCACAGGCCACATTCACGT-3′<br>REV 5′- GTTCACAAATCAGCACCAAGC-3′
Survivin	*BIRC5*	FOR 5′- GGACCACCGCATCTCTACAT-3′<br>REV 5′- GTTGCGCTTTCCTTTCTGTC-3′
Prominin 1	*PROM1*	FOR 5′- TTGTGGCAAATCACCAGGTA-3′<br>REV 5′- TCAGATCTGTGAACGCCTTG-3′
SRY-box transcription factor 2	*SOX2*	FOR 5′- CACATGAAGGAGCACCCGGATTAT-3′<br>REV 5′- GTTCATGTGCGCGTAACTGTCCAT-3′
Nestin	*NES*	FOR 5′-AACAGCGACGGAGGTCTCTA-3′<br>REV 5′-TTCTCTTGTCCCGCAGACTT-3′
Glial fibrillary acidic protein	*GFAP*	FOR 5′- CTGTCCCTAGGTCAGCTTGC-3′<br>REV 5′- GATGTGGAGGGCGATGTAGT-3′
Notch receptor 1	*NOTCH 1*	FOR 5′- GTGACTGCTCCCTCAACTTCAAT-3′<br>REV 5′- GGAATGAAGTCCAAACCGGTG-3′
Notch receptor 2	*NOTCH 2*	FOR 5′- CACCCCAGCTGCTACTCACA -3′<br>REV 5′- GCCAACCCAGCCTGCAT-3′
Notch receptor 3	*NOTCH 3*	FOR 5′- CCTGTCTTCCTGGGTTTGAG -3′<br>REV 5′- CAGAACTGGCCTGTGCACTC-3′
Notch receptor 4	*NOTCH 4*	FOR 5′- CCAACCCTGCGATAATGCGAG -3′<br>REV 5′- AGTCATCCGTTGAGACCCTGC-3′
Glyceraldehyde 3-phosphate dehydrogenase	*GAPDH*	FOR 5′- CCCACTCCTCCACCTTTGAC-3′<br>REV 5′- TGTTGCTAGCCAAATTCGTT-3′
